# Vitamins as primary or adjunctive treatment in infertile men with varicocele: A systematic review

**DOI:** 10.1080/2090598X.2021.1932124

**Published:** 2021-05-29

**Authors:** Georgios Tsampoukas, Kristiana Gkeka, Athanasios Dellis, Dominic Brown, Antigoni Katsouri, Ahmed Alneshawy, Mohamad Moussa, Athanasios Papatsoris, Noor Buchholz

**Affiliations:** a U-merge Ltd. (Urology for Emerging Countries), London, UK; b Department of Urology, Princess Alexandra Hospital, Harlow, UK; c Department of Urology, General Hospital of Patras, Patras, Greece; d Department of Urology, Aretaieion Academic Hospital, Athens, Greece; e Department of Pharmacy, University of Patras, Patras, Greece; f Al Zahraa Hospital, University Medical Center, Lebanese University, Beirut, Lebanon; g Second Department of Urology, University Hospital of Athens, Athens, Greece

**Keywords:** Male infertility, vitamins, semen parameters, pregnancy rates, varicocele

## Abstract

**Objective:**

To investigate the usage and the efficacy of vitamins as primary or adjuvant treatment in infertile men with varicocele.

**Methods:**

A systematic search in PubMed, the Medical Literature Analysis and Retrieval System Online (MEDLINE) and Cochrane Library with the terms (varicocele) AND (vitamins) was performed. We searched for studies: a) reporting the administration of vitamins (individually or as part of a complex) in men with varicocele and infertility, b) primarily or adjuvant to invasive treatment, and c) reporting the impact on semen parameters and/or pregnancy rates. Exclusion criteria were animal, adolescent and non-English studies, grey literature and trials reporting abstracts only.

**Results:**

Seven studies were identified eligible for qualitative analysis. All studies were randomised except one (case series). Vitamins were administered dominantly as part of antioxidant complex and only two studies used vitamins (C and E, respectively) as sole agent. In two studies, vitamin monotherapy resulted in improvement in semen quality, but the effect on pregnancy rates is unknown. One study reported no efficacy of adjuvant multivitamin treatment after embolisation in terms of both semen quality and pregnancy rates. Finally, four studies reported a positive effect of vitamins on semen parameters after varicocelectomy, but the effect on pregnancy rates is conflicting; one study reported improved pregnancy rates with adjuvant treatment, two studies did not evaluate the pregnancy rates, and in one study the outcome was unclear due to missing data.

**Conclusions:**

Vitamins have been used mostly as part of an antioxidant panel for the management of infertile men with varicocele. Most studies have found a positive impact on semen parameters in selected men with varicocele and infertility, as primary or adjuvant treatment. However, the clinical benefit of vitamins administration on pregnancy rate is under-evaluated and should be the target of future research.

## Introduction

Varicocele is regarded as one of the most frequent causes of male infertility. It is defined as the abnormal dilatation of the veins within the pampiniform plexus associated with venous reflux [1]. It can be found in a significant number of the healthy male population (up to 20%), its prevalence increases with age and is strongly associated with secondary infertility [2]. Varicocele is thought to affect fertility by impairing sperm parameters, including density, motility and morphology, and by decreasing testicular volume [3]. Interventional treatment is regarded as the cornerstone of management, as it provides high success rates in terms of improvement of dyspermia and pregnancy rates in both primary and secondary infertility [4–6]. In the modern era, new insights into the hypothesis of elevated oxidative stress have broadened new routes in the management of this condition [7]. This hypothesis concentrates the highest interest, as it is common ground of multiple studies. In infertile men with varicocele, excessive oxidative stress has been found in their seminal plasma, findings that might also reflect the severity of varicocele and the location [8–10]. These findings follow the rapidly expanding area of research regarding the role of dietary supplements in male infertility, where countless agents have already been tested with various efficacy [11]. Moreover, the overlapping between other causative factors and varicocele makes the evaluation of adjunctive or alternative treatments enticing. Towards that direction, primary or supplementary treatments with antioxidant agents after varicocelectomy have been investigated and the results are promising, as patients may enjoy improvements in their semen parameters that might be helpful for the final outcome [12,13]. Vitamins represent a nutritional category of special interest, as they carry both antioxidant capacity and other unique characteristics involved with body health. They have already been used as potential agents in various fields, including the cardiovascular, mental and urological systems [14–16]. As a result, along with their multiple benefits, vitamins are promising conservative agents for the management of male infertility, as they counteract the environmental, lifestyle and nutritional factors affecting the male fertility capacity and this ability seems to include infertile men with varicocele [17,18]. However, knowledge of the exact type of vitamin needed, dosage and duration of treatment, optimal patient selection criteria, cost-effectiveness and the optimal combination of agents is still lacking. In the present review, we investigate the usage of vitamins in the management of infertile men with varicocele and we discuss the current evidence regarding their role in the condition’s management.

## Methods

### Study retrieval

We performed the present systematic review according to the Preferred Reporting Items for Systematic Reviews and Meta-Analyses (PRISMA) guidelines [19]. A search in PubMed, Cochrane library and the Medical Literature Analysis and Retrieval System Online (MEDLINE) was performed using the terms (*varicocele)* AND (*vitamins)*. Also, we ran a secondary search in PubMed and MEDLINE using the terms *(varicocele)* AND *(antioxidants)*, in order to identify relevant studies missed in our primary search. No time limits were applied.

### Inclusion criteria

Inclusion criteria were adult studies reporting the outcomes of vitamins in the management of infertile, adult men with clinically or ultrasonographically diagnosed varicocele of any grade.

### Exclusion criteria

Exclusion criteria were animal, adolescent and non-English studies, grey literature, case reports, editorials and trials reporting abstracts only.

### Data extraction

**Figure 1.**
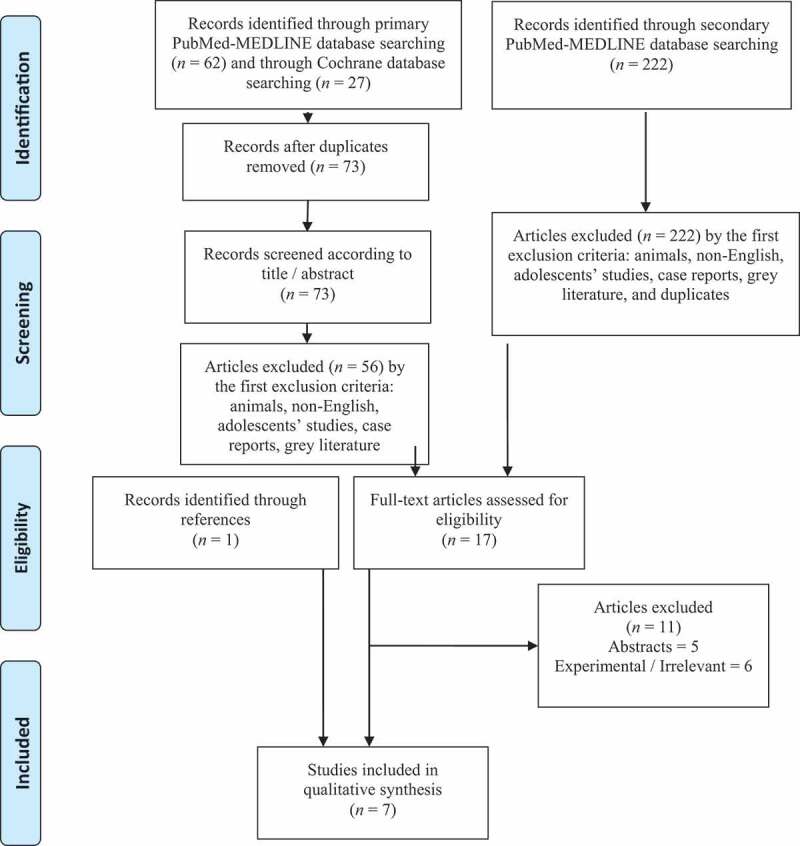
PRISMA flow chart

Data of each study were extracted independently by two investigators. The demographic and clinical characteristics of included studies were documented. The primary outcomes of the studies were semen quality (semen count or concentration, motility, morphology, according to WHO guidelines and sperm DNA damage) and pregnancy rates. The strategy of our search was according to the PRISMA approach and is illustrated in Figure 1.

### Assessment of methodological quality

The risk of bias stratification of the randomised studies was calculated with the Risk of Bias 2 tool by the Cochrane Scientific Committee. The assessment is presented in Table 1 [20,21,23 –26]. The risk of bias of the case-series study was not calculated as by definition the study was exposed to bias due to the lack of a control.

**Table 1.** Risk of bias of randomised studies

	Randomisation process	Deviation from intended interventions	Missing outcome data	Measurement of the outcome	Selection of the reported result	Overall
Paradiso Galatioto *et al*. [23]	(?)	(+)	(+)	(+)	(+)	(?)
Cyrus *et al*., 2015 [24]	(?)	(+)	(+)	(+)	(+)	(?)
Ener *et al*., 2016 [26]	(+)	(+)	(?)	(+)	(?)	(?)
Busetto *et al*., 2018 [20]	(+)	(+)	(+)	(+)	(+)	(+)
Kızılay *et al*., 2019 [25]	(+)	(+)	(+)	(+)	(+)	(+)
Ardestani Zadeh *et al*., 2019 [21]	(+)	(+)	(+)	(+)	(+)	(+)

Low risk: (+); Some concerns: (?); High risk: (–)

## Results

In the primary search, we identified 62 and 27 articles while searching the PubMed-MEDLINE database and the Cochrane Library, respectively. The secondary search revealed 222 articles but none of them were identified for qualitative analysis. We assessed a total of 17 full-text articles for eligibility and we excluded 11 of them, as they did not meet the inclusion criteria. While, another article identified through the reference lists, was added. Therefore, a total of seven studies were included in the qualitative synthesis, reporting the outcomes of vitamins alone or as a part of a regime in infertile patients with varicocele. The age of the studies’ participants ranged between 18 and 50 years. Regarding the type of study, there were six randomised controlled trials (RCTs) and one prospective case series. From the seven studies included, three of them evaluated the effect of vitamins on semen parameters [14,16,19] and the remaining four reported the outcomes of vitamins on semen parameters and pregnancy rates [13,15,17,18]. Moreover, five studies compared a panel of vitamins and antioxidants as adjuvant treatment to interventions alone (embolisation, one study; varicocelectomy, four studies), while two studies reported outcomes for the efficacy of vitamins as monotherapy. The data are presented in Table 2 [20–26].

**Table 2.** Vitamins and varicocele; synthesis of evidence

Reference	Age, years	Study design	Level of evidence/populations	Treatment regimen	Endpoints	Outcome	Comments
Paradiso Galatioto *et al*. [23]	23–36	RCT	42 infertile men with colour Doppler ultrasound Grade III, IV, and V varicocele, remaining persistently oligospermic 6 months after retrograde embolisation Adjuvant treatment (Group 1, *n*= 20) vs no adjuvant treatment (Group 2, *n*= 22)	Vitamin C, E, A, D, complex B (B2, B3, B5, B6, B7, B12), calcium, magnesium, phosphate, iron, manganese, copper and zinc once daily for 3 months	Report of improvement in semen parameters and pregnancy success during an observational period of 12 months	In favour of adjuvant treatment in terms of semen parameters improvement in normal sperm count, but not statistically significant in motility and morphology – no significant impact on pregnancy rates Total sperm count, × 10^6^/mL Before treatment: 14.42 After treatment: 35.28 *P* = 0.009	A significant difference in the duration of infertility was observed between two groups; treated group had significantly longer duration infertility in comparison to controls No significant side effects to antioxidant therapy
Gual-Frau *et al*., 2015 [22]	Unknown	Prospective case series	20 infertile men with clinical Grade I varicocele and asthenoteratozoospermia receiving antioxidant treatment	L-carnitine, vitamin C, CoQ10, vitamin E, vitamin B9, vitamin B12, zinc, and selenium per day for 3 months	Report of improvement in DNA fragmentation and pregnancy success	Positive effect of treatment on sperm count and DNA fragmentation; two couples achieved pregnancy Sperm DNA fragmentation, % Before treatment: 30.38 ± 13.32 After treatment: 23.65 ± 7.5 *P* = 0.02 Total sperm count, × 10^6^/mL Before treatment:183.64 ± 153.54 After treatment: 299.85 ± 275.45 *P* = 0.04	Antioxidant treatment did not improve the primary semen abnormality significantly Absence of control arm; small sample size
Cyrus *et al*., 2015 [24]	27.6 ± 5.3	RCT	115 infertile men with clinical Grade II and III varicocele and dyspermia Group receiving adjuvant treatment after varicocelectomy (Intervention Group, *n*= 46) vs no adjuvant treatment (Placebo group, *n*= 69)	Vitamin C twice daily for 3 months	Report of improvement of semen parameters	Significant improvement with adjuvant treatment in terms of motility and morphology Motility, % Intervention group:54.5 ± 18.3 Placebo group:44.9 ± 21.4 *P* = 0.041 Morphology, % Intervention group:75.3 ± 13.1 Placebo group:67.5 ± 16.4 *P* < 0.001	The surgical approach was open inguinal method (Ivanissevich) Grade III varicoceles were dominant (Intervention group: 80.5%; Placebo group: 85.5%) Intervention group were younger; vitamin C contributed to the improvement of motility and morphology regardless of age (*P* = 0.044 and *P* = 0.001, respectively) A significant number of cases had decline in sperm count, motility and normal morphology
Ener *et al*., 2016 [26]	25.8 ± 4.6	RCT	45 infertile men with clinical varicocele Adjuvant vitamin E after varicocelectomy for 12 months (Group 1, *n*= 22) vs no adjuvant treatment (Group 2, *n*= 23)	Vitamin E daily for 12 months	Report of semen quality and spontaneous pregnancy success	No significant benefit of adjuvant treatment on semen parameters; pregnancy rate was 22% in 12 months regardless of vitamin E supplementation Sperm count, × 10^6^/mL Preoperative:36.5 ± 29.8 Postoperative 12 months: 58.6 ± 20.2 *P* = 0.121 Motile sperm, % Preoperative: 54.1 ± 20.4 Postoperative 12 months: 59.3 ± 16.2 *P* = 0.698	Subinguinal varicocelectomy with binocular loops No comment on grade No comment of impact of vitamin E on pregnancy success
Busetto *et al*., 2018 [20]	18–50	RCT	45 infertile patients with oligo- and/or astheno- and/or teratozoospermia with Grade I–III varicocele Supplementary treatment (Supplementation, *n*= 21) vs placebo (Placebo, *n*= 24)	L-carnitine, acetyl-L-carnitine, fumarate, fructose, CoQ10, vitamin C, zinc, folic acid and vitamin B12 twice daily for 6 months	Report of improvement of semen parameters	Superiority of vitamins administration in terms of improvement in total sperm count, progressive and total motility, morphology Supplemented group: Total sperm count, × 10^6^/mL: Baseline: 96.3 ± 36.1 Final visit: 158.8 ± 90.1 *P* < 0.001 Progressive motility, %: Baseline:23.1 ± 5.2 Final visit: 27.4 ± 7.2 *P* = 0.015 Total motility, %: Baseline: 31.5 ± 8.1 Final visit: 37.5 ± 7.1 *P* = 0.007	Pregnancy success was not the endpoint of the study (2 pregnancies occurred −1 in the supplementation group and 1 in the placebo group) 4 patients developed mild, probably associated side effects (nausea, reflux, headache, vertigo)
Kızılay *et al*., 2019 [25]	32.86 ± 3.14	RCT	93 infertile men with clinical Grade I–III varicocele and oligo-and/or astheno- and/or teratozoospermia Group receiving adjuvant treatment after varicocelectomy (Group 1, *n*= 62) vs no adjuvant treatment (Group 2, *n*= 28)	L-carnitine fumarate, acetyl-L-carnitine HCl, fructose, citric acid, vitamin C, zinc, folic acid, selenium, CoQ10, vitamin B12 twice daily for 6 months	Report of improvement in semen parameters and pregnancy rates	Superiority of adjuvant treatment in terms of sperm count, morphology, concentration, all kinds of motility; higher pregnancy rate Group 1: Total sperm count, × 10^6^/ejaculate: Baseline: 22.09 ± 3.89 Postop.: 32.22 ± 6.11 *P* < 0.001 Sperm concentration, × 10^6^/mL: Baseline: 8.24 ± 1.88 Postop.: 14.12 ± 2.11 *P* = 0.037 Normal morphology, %: Baseline: 1.89 ± 0.45 Postop.: 3.32 ± 0.3 *P* = 0.041 Total motility, %: Baseline: 30.19 ± 5.16 Postop.: 38.83 ± 10.4 *P* = 0.018 Progressive motility, %: Baseline: 16.25 ± 3.2 Postop.: 26.08 ± 7.62 *P* < 0.001	Microsurgical subinguinal varicocelectomy 9 patients presented mild, related to medication side-effects, controlled by palliative treatment (nausea, gastro-oesophageal reflux)
Ardestani Zadeh *et al*., 2019 [21]	30.37 ± 5.38	RCT	60 infertile men with clinical varicocele Grade I–III Supplementary treatment (Supplement group, *n*= 30) vs no treatment (varicocelectomy alone, control group, *n*= 30) after varicocelectomy	Vitamin E, selenium, folic acid once daily for 6 months	Report of improvement in semen parameters	Superiority of adjuvant treatment in terms of sperm count and motility Supplemented group: Sperm concentration, × 10^6^/mL: Preop.: 35.92 ± 23.14 Postop.: 41.26 ± 24.52 *P* = 0.021 Motility readings, %: Preop.: 46.45 ± 16.02 Postop.: 50.29 ± 15.14 *P* = 0.003	Subinguinal varicocelectomy with binocular loops Pregnancy success was not reported

Postop.: postoperative; Preop.: preoperative.

### Vitamins as monotherapy for infertile patients with varicocele

Two studies were identified reporting the performance of vitamins as monotherapy in infertile patients with varicocele. In the first study, 20 patients with clinical Grade I varicocele and asthenoteratozoospermia were given a complex of vitamins (L-carnitine, vitamin C, coenzyme Q10 [CoQ10], vitamin E, vitamin B9, vitamin B12, zinc and selenium) per day for a follow-up of 3 months. The authors observed that the sperm DNA fragmentation was significantly reduced after treatment, but the semen parameters were not statistically significantly improved except for the total sperm count. Two of the couples achieved pregnancy during the observational period [22]. A double-blind, placebo-controlled study reported the efficacy of multivitamins administration (L-carnitine, acetyl-L-carnitine, fumarate, fructose, CoQ10, vitamin C, zinc, folic acid and vitamin B12) twice daily for 6 months in 45 infertile men with varicocele Grade I–III and oligoasthenoteratozoospermia. The authors randomised the patients into two groups: the first receiving the multivitamins complex (*n* = 21) and the second receiving the placebo (*n* = 24). The patients were followed after 6 months with a repeat spermiogram. At the end of the study, the supplemented group had a significant improvement in their total sperm count, and progressive and total motility. Although the pregnancy rate was not an endpoint of the study, during the follow-up period two pregnancies occurred [20].

### Vitamins as adjuvant treatment after embolisation

One study reported the efficacy of adjuvant multivitamin treatment after embolisation. Paradiso Galatioto *et al*. [23] investigated the role of adjuvant multivitamins treatment in 42 infertile men with clinical varicocele Grade III–V and persistent oligospermia after retrograde embolisation. The treated group of 20 men received a complex of N-acetyl-cysteine and vitamins and minerals once daily for 3 months (vitamin C, vitamin E, vitamin A, thiamine, riboflavin, piridoxin, nicotinamide, pantothenate, biotin, cyanocobalamin, ergocalciferol, calcium, magnesium, phosphate, iron, manganese, copper and zinc), while the other 22 men did not receive antioxidant therapy (control group). Both groups were followed with a spermiogram at 3 months and the pregnancy rate was recorded during the12-month observation period after treatment withdrawal. Treated patients had increased chances of achieving a normal sperm count compared to the untreated ones, but the benefit was not significant, neither for motility nor morphology. Also, the authors found no impact of adjuvant treatment on the spontaneous pregnancy rate.

### Vitamins as adjuvant treatment after varicocelectomy

Four studies were identified reporting the potential effect of vitamins after varicocelectomy. In the first study, 115 infertile men with varicocele Grade II–III and dyspermia were randomised in two groups after varicocelectomy (Ivanissevich technique). Group 1 comprised 46 patients who received 250 mg vitamin C twice daily for 3 months after surgery, whereas Group 2 patients received placebo. The authors found that the administration of vitamin C was associated with increased motility and morphology, but there was no significant impact on sperm count. The pregnancy rates were not recorded [24]. In the second study, 90 patients were divided with simple random allocation in two groups after varicocelectomy. In the first group, 62 patients received antioxidant treatment, containing L-carnitine fumarate, acetyl-L-carnitine HCl, fructose, citric acid, vitamin C, folic acid, zinc, selenium, CoQ10 and vitamin B12, twice daily for 6 months, whereas the second group (29 patients) did not receive any treatment. In the first group the improvement in total sperm count, sperm concentration, sperm count with normal morphology, total motility, progressive motility as well clinical pregnancy rate was significantly higher (29%) [25]. The two remaining studies focussed on the efficacy of vitamin E on the improvement of semen parameters after varicocelectomy. Ener *et al*. [26] randomised 45 infertile patients who underwent varicocelectomy into two groups: 22 of them received daily 600 mg vitamin E orally after the varicocelectomy for 12 months, while the remaining 23 patients comprised the control group and received no treatment. Despite some improvement in semen parameters, the authors found no merit for the adjuvant role of vitamin E in sperm count and in the percentage of motile sperm. Therefore, they concluded that the treatment of infertile patients with varicocele should primarily be focussed on surgery. In another study, vitamin E along with selenium and folic acid was tested as an adjuvant treatment after surgery. A total of 60 patients were randomised into two groups after subinguinal varicocelectomy: 30 patients received a combination of the above once daily for 6 months whereas the rest received no treatment. The supplemented group showed a significantly higher sperm concentration and motility readings after 6 months of treatment. The pregnancy rates were not recorded [21].

## Discussion

The most promising outcome of the above studies was the improvement of some specific semen parameters and especially motility. We believe that these findings are meaningful. This way, patients with varicocele enjoy increases in their total motile sperm count (TMSC). The role of TMSC (calculated by multiplying the volume of the ejaculate by the sperm concentration by the proportion of progressive motile sperm divided by 100%) has been highlighted as a strong predictive marker of spontaneous pregnancy rates and post-assisted reproductive treatments outcomes [27,28]. Therefore, vitamins might be used in the appropriate clinical context if a further boost in the outcome of surgery is needed or intervention has not been decided yet. However, no definitive conclusions can be drawn as the studies were quite heterogenic, different panels of agents were used, and only a few studies studied the efficacy of vitamins as monotherapy. Also, the cost-effectiveness of the regimes used is unknown, whereas the main effect measure of outcome in the studies was semen parameters, which is a surrogate marker of male fertility. On the other hand, pregnancy rates as the most reliable tool of fertility capacity were not systematically examined.

Some comments are noteworthy regarding the individual capacity of vitamins in the management of varicocele-induced infertility. We are inclined to highlight the role of vitamins C and E, which were used as single agents and are the common ground in most studies. The concentration of vitamin E in seminal plasma has been associated with the antioxidant capacity of the semen and the percentage of motile spermatozoa [29,30]. In idiopathic male infertility, the combination of vitamin E and clomiphene citrate has been shown to be effective in infertile men with oligoasthenospermia in terms of improvement in semen parameters [31]. In experimental studies, vitamin E has been shown to improve the endocrine function and the spermatogenesis process in the varicocelised testis after co-administration with testosterone or dexamethasone [32,33]. Therefore, it seems that vitamin E works more efficiently as a supplementary to other agents and this should be taken into consideration in patients with varicocele as well. However, the optimal combination is unknown. Ascorbic acid has also been studied as a potent antioxidant factor improving semen parameters and optimising the chances of fatherhood [34]. It has been associated with improvements in semen quality in a dose-dependent manner in smokers [35], whereas the main benefits seem to lie on motility [36]. Furthermore, meaningful effects of vitamin C administration on DNA quality and mRNA levels on the semen of patients with recurrent pregnancy loss have been observed [37]. In patients with varicocele, increased oxidative stress markers have been found to be negatively associated with the concentration of vitamin C in the internal spermatic vein [38]. As aforesaid, the adjuvant administration of vitamin C is associated with improvements in motility and morphology [24]. Such meaningful observations might further assist the clinician to optimise the next step after surgery in men with varicocele who seek fatherhood and might need to resort to assisted-reproduction modalities [39].

Also, vitamin D (ergocalciferol) has also been evaluated in the pathophysiology of male infertility, as infertile men have been found with vitamin D deficiency more often than fertile men [40]. Furthermore, vitamin D has a positive effect on the intracellular calcium concentration of spermatozoa, which facilitates sperm motility and the acrosome reaction [41]. In a randomised study, administration of vitamin D was meaningful in the subgroup of oligospermic men, as it increased their chances for a live birth compared with placebo, albeit the effect was not apparent in the spermiogram [42]. If these findings are meaningful for patients with varicocele, it needs to be clarified in future studies.

Vitamins of complex B represent a broad family of individual vitamins with promising potential in male infertility. In men with oligoasthenoteratozoospermia, the administration of vitamins B9 and B12 as part of a dietary complex has increased the chances to achieve normospermia, whereas there was a significant boost to spontaneous pregnancy success comparing to placebo [43]. In varicocelised rats, the administration of vitamin B significantly increased sperm parameters, chromatin integrity and lipid peroxidation compared to vitamin E [44]. As the evidence seems to be sparse, the B vitamins might be part of the empirical treatment of infertile men, but no specific recommendations can be made.

Finally, retinoic acid (vitamin A) is regarded as a crucial factor of adult spermatogenesis; germ cell development is mediated by the molecule through a rambling pathway in a process that is coordinated also by FSH and testosterone [45–47]. Subsequently, the disruption of retinoic acid receptor alpha (RARα) function may result in primary male subfertility. In patients with varicocele, it has been shown that RARα expression is significantly reduced, whereas the response to vitamin A treatment is also altered [48]. Also, treatment with vitamin A may enhance superoxide dismutase and glutathione transferase activities, and decrease the oxidative stress in the semen of patients with varicocele [49]. In a study by Paradiso Galatioto *et al*. [23] the administration of vitamin A along with a complex of multivitamins and minerals resulted in an increase in the sperm count, but there was no effect on pregnancy rates. The interpretation of these results is difficult as the patients represented a specific subgroup of men with longstanding infertility who were oligospermic and had previous embolisation. If any benefit exists, the optimisation of the selection criteria for vitamin A treatment as a primary or adjuvant agent needs to be clarified with future studies.

There may be some possible limitations of the present review. Firstly, the effect of vitamins supplementation on pregnancy rates is uncertain at best, as this was not the primary endpoint in all studies. Also, most studies have used vitamins as part of a complex of antioxidants and the host of combined agents used does not permit conclusions about individual agents. Furthermore, dosages and regimes varied resulting in lack of reproducibility and uncertain cost-effectiveness. It is noteworthy as well, that although the studies evaluated the treatment effect of vitamins, possible deficits in the serum or semen of the patients were not examined. A comparison of subgroups of responders vs non-responders in that context would be useful. Moreover, it is difficult to define the safety profile of agents in the above studies, as patients developing symptoms side-effects were systematically excluded [21,24]. Finally, although the performance of empirical treatments in male infertility is reasonable, it seems mandatory for future research to dictate a more patient-tailored approach. It is a fact that despite the established role of oxidative stress, the administration of antioxidant factors does not always benefit the human body originating the so-called ‘antioxidant paradox’ phenomenon [50]. This observation along with the harming results of reductive stress have raised concerns for more accurate therapies and specific guidelines in patients with infertility [51].

## Conclusion

The role of vitamins in the management of patients with varicocele looks promising, but the primary endpoint of pregnancy rate still needs to be evaluated. The physiological significance of the agents is noteworthy, but future studies need to clarify the specific clinical context and optimum regimens in which the vitamins could be used. To date, vitamin treatment might be considered as a supplementary tool for the management of patients with varicocele to further ameliorate their semen parameters.
